# Hemoconcentration and predictors in Shiga toxin-producing *E. coli*-hemolytic uremic syndrome (STEC-HUS)

**DOI:** 10.1007/s00467-021-05108-6

**Published:** 2021-05-27

**Authors:** Sebastian Loos, Jun Oh, Laura van de Loo, Markus J. Kemper, Martin Blohm, Raphael Schild

**Affiliations:** 1 University Medical Center Hamburg-Eppendorf, University Children’s Hospital, Martinistrasse 52, 20246 Hamburg, Germany; 2 University Children’s Research@Kinder-UKE, University Medical Center Hamburg-Eppendorf, Hamburg, Germany; 3 Department of Pediatrics, Asklepios Klink Nord, Hamburg, Germany

**Keywords:** Children, HUS, EHEC, STEC, Hemoconcentration, Predictors, Outcome

## Abstract

**Background:**

Hemoconcentration has been identified as a risk factor for a complicated course in Shiga toxin-producing *E. coli*-hemolytic uremic syndrome (STEC-HUS). This single-center study assesses hemoconcentration and predictors at presentation in STEC-HUS treated from 2009–2017.

**Methods:**

Data of 107 pediatric patients with STEC-HUS were analyzed retrospectively. Patients with mild HUS (mHUS, definition: max. serum creatinine < 1.5 mg/dL and no major neurological symptoms) were compared to patients with severe HUS (sHUS, definition: max. serum creatinine ≥ 1.5 mg/dL ± major neurological symptoms). Additionally, predictors of complicated HUS (dialysis ± major neurological symptoms) were analyzed.

**Results:**

Sixteen of one hundred seven (15%) patients had mHUS. Admission of patients with sHUS occurred median 2 days earlier after the onset of symptoms than in patients with mHUS. On admission, patients with subsequent sHUS had significantly higher median hemoglobin (9.5 g/dL (3.6–15.7) vs. 8.5 g/dL (4.2–11.5), *p* = 0.016) than patients with mHUS. The product of hemoglobin (g/dL) and LDH (U/L) (cutoff value 13,302, sensitivity 78.0%, specificity of 87.5%) was a predictor of severe vs. mild HUS. Creatinine (AUC 0.86, 95% CI 0.79–0.93) and the previously published score hemoglobin (g/dL) + 2 × creatinine (mg/dL) showed a good prediction for development of complicated HUS (AUC 0.87, 95% CI 0.80–0.93).

**Conclusions:**

At presentation, patients with subsequent severe STEC-HUS had a higher degree of hemoconcentration. This underlines that fluid loss or reduced fluid intake/administration may be a risk factor for severe HUS. The good predictive value of the score hemoglobin (g/dL) + 2 × creatinine (mg/dL) for complicated HUS could be validated in our cohort.

**Graphical abstract:**

**Figure:**
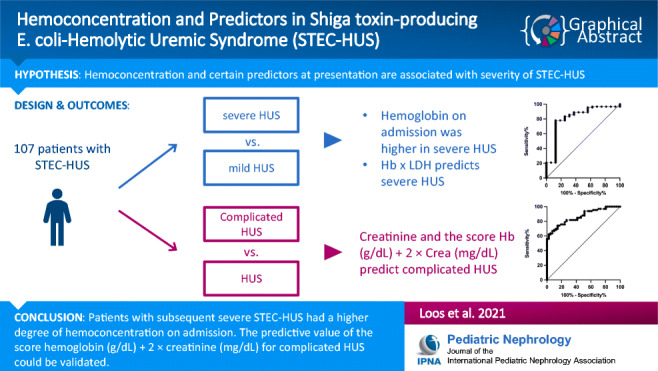
A higher resolution version of the Graphical abstract is available as Supplementary Information.

**Supplementary Information:**

The online version contains supplementary material available at 10.1007/s00467-021-05108-6.

## Introduction

Intestinal infections with Shiga toxin-producing *Escherichia coli* (STEC) can lead to (hemorrhagic) colitis and, usually after several days of gastrointestinal prodomi, be complicated by hemolytic uremic syndrome (HUS) [1]. Shiga toxin (Stx) plays a key role in the pathogenesis of STEC-HUS. After ingestion and intestinal infection, Stx is translocated from the gut into the bloodstream. Once in the bloodstream, Stx induces systemic proinflammatory and prothrombotic pathways [2]. For the broad spectrum of other causes of HUS, we refer to recent review articles [3, 4].

STEC-HUS mainly affects children below the age of 5 years and is defined by non-immune hemolytic anemia, thrombocytopenia, and acute kidney injury (AKI) in the context of STEC infection [1]. Due to the preceding several days of gastroenteritis, most patients are dehydrated and hemoconcentrated at the onset of STEC-HUS. Thus, beside the direct effects of Stx, a prerenal component might contribute to AKI [5]. STEC-HUS can affect multiple organs, e.g., the kidney and the brain. This can result in severe neurological complications. Therapy of STEC-HUS is mainly supportive including fluid management, transfusions, treatment of hypertension, kidney replacement therapy (KRT), and treatment of neurological complications. Most patients recover completely. However, the case fatality rate of STEC-HUS is around 1–5%. Additionally, follow-up data from Germany and Austria show relevant sequelae of STEC-HUS with chronic kidney disease (CKD) ≥ stage 2 in 4–7% and potentially major neurological sequelae [6, 7]. Thus, the management of STEC-HUS has to be further optimized.

Several easily accessible clinical parameters have been evaluated or identified as prognostic markers in STEC-HUS. This includes parameters of thromboinflammation (e.g., leukocytosis [8–10], elevated D-dimers [11], complement activation [12]), urea [12], sodium [13], and lactate dehydrogenase (LDH) [14–16].

Additionally, high hematocrit or hemoglobin levels as surrogate parameters of hemoconcentration have been associated with developing STEC-HUS or a more severe course of STEC-HUS [5, 9, 13, 15, 17–20]. Based on these findings, Ardissino et al. suggested a score (hemoglobin (g/dL) + 2 × creatinine (mg/dL) on admission) to identify patients at high risk for a severe course. The score was moderately predictive for a complicated course of HUS (AUC 0.75, 95% CI 0.67–0.85), defined by the outcome death or CKD stage 2–5 [18]. Recently, Lin et al. validated the score for the end-points dialysis, neurologic complications, respiratory failure, and death (AUC 0.77, 95% CI 0.68–0.87) [21].

Several studies have shown that volume expansion with infusion therapy improves the outcome. Early initiation of volume expansion during the gastrointestinal phase reduces the risk to develop HUS at all [15] or oligoanuric HUS [5, 22, 23]. Volume expansion in established STEC-HUS mitigates the course of the disease, eventually reducing the need for dialysis and the rate of neurological complications. In a cohort of 38 patients treated by infusion therapy and compared to historical controls, Ardissino et al. showed that neurological involvement (8% vs. 24%, *p* = 0.06) and KRT (26% vs. 58%, *p* = 0.01) could be reduced [24]. Recently, Bonany et al. published a similar study showing a reduced need for dialysis in patients treated with fluid infusion (*n* = 16, dialysis in 12.5%) compared to historical controls treated by fluid restriction (*n* = 19, dialysis in 47%) [25].

Due to the fact that some patients are severely dehydrated and hemoconcentrated at the beginning of STEC-HUS with a subsequent apparent normal hemoglobin level, it has also been questioned whether the classical definition of STEC-HUS should be applied at all stages of the disease and if this definition might reduce the chance to identify patients with STEC-HUS early [15, 26]. Additionally, not all patients do fulfill all three abovementioned classical criteria for HUS at onset or even during the course of HUS [26, 27]. Especially in milder courses of HUS, the AKI might be accompanied by only a slight increase of creatinine.

Based on the hypothesis that a severe course of the disease is associated with more hemoconcentration, the aim of this study was to evaluate the initial hemoconcentration and predictors for the course of HUS in our single-center cohort.

## Methods

### Patients

Medical records of all patients treated for STEC-HUS at our center between 2009 and 2017 were analyzed retrospectively. Patients with atypical/complement-, pneumococcal-, and pertussis-associated HUS (*n* = 8) were excluded. Some data of 33 patients included in this study have been published previously [28].

Microbiologic analysis for STEC infection was based on the local protocol including (enriched) stool cultures for STEC and immunoassay or polymerase chain reaction (PCR) for detection of Stx.

The following clinical definitions were applied:

- *HUS*: hemolytic anemia (minimum hemoglobin below the lower limit of the normal range), thrombocytopenia (minimum thrombocytes < 150 × 10^9^/L), and serum creatinine above the upper limit of the age-dependent normal range. Two patients had minimum thrombocytes > 150 × 10^9^/L but showed platelet consumption.
- *Severe HUS (sHUS)*: HUS with a maximum serum creatinine ≥ 1.5 mg/dL ± major neurological symptoms (definition: impairment of consciousness (stupor or coma), epileptic seizures, focal neurological deficits (e.g., paresis), and/or visual disturbances (double or blurry vision)) during the course of the disease.
- *Mild HUS (mHUS)*: HUS with a maximum serum creatinine < 1.5 mg/dL (reflecting the KDIGO-criteria for the AKI stage 1 [29]) and absence of major neurological symptoms.

The local ethical committee approved the study (PV3975, WF-016/19).

### Statistics

Descriptive statistics are presented for continuous variables (median and range) and for categorical variables (number and percentage). Continuous variables were compared using the Mann–Whitney *U* test for two groups or the Kruskal–Wallis test followed by the Dunn procedure for multiple groups. A receiver operating characteristic (ROC) curve was plotted for specific laboratory parameters. Pearson’s *χ*^2^ test was used for categorical data. *P* values < 0.05 were considered statistically significant. Data were analyzed using PRISM (Version 8, GraphPad, USA).

## Results

### Cohort

A cohort of 107 patients with STEC-HUS was analyzed (females: 58 (54%), males: 49 (46%)). The median age was 4.0 years (0.5–16.6). STEC or Stx in the stool could be detected in 79 (74%) patients by culture and/or PCR/immunoassay.

Major neurological symptoms occurred in 35 (33%) patients. Mortality was 3/107 (3%) due to neurological complications (cerebral bleeding, edema).

### Mild HUS vs. severe HUS

Patients were divided into two groups according to the definitions given above.

Sixteen (15%) patients had mild HUS (mHUS). In all patients, maximum serum creatinine was < 1.5 mg/dL. In 11/16 patients, it was even < 1.0 mg/dL. None received dialysis.

Ninety-one (85%) patients had severe HUS (sHUS). In total, KRT was performed in 61 (57%) patients (61/91 (67%) in the sHUS group). The median duration of dialysis was 10 days in those where dialysis could be terminated. Three patients were on dialysis at discharge. Three patients in the sHUS group had a max. creatinine < 1.5 mg/dL but major neurological symptoms.

### Initial hemoconcentration in sHUS vs. mHUS

Patients with mHUS were admitted later after onset of symptoms (diarrhea, vomiting, abdominal pain, and/or fever) than patients with sHUS (Table 1). Compared to mHUS cases, patients with sHUS had a significantly higher hemoglobin (Table 1) and hematocrit (27.3% (10.0–44.2) vs. 23.6% (12.4–33.5), *p* = 0.016) on admission. This was even significant after adjustment by calculation of absolute and relative (percentage) hemoglobin reduction from the lower limit of the age-dependent reference values (data not shown).

**Table 1:** Demographic and laboratory data (median, range) of 107 patients. To convert values for creatinine to μmol/L multiply by 88.4

		Mild HUS (*n* = 16)	Severe HUS (*n* = 91)	*p*
	Age, years	3.0 (0.8–16.5)	4.8 (0.5–16.6)	0.07
	Males, *n* (%)	7 (44)	42 (46)	0.86
	Duration of symptoms^a^ on admission, days	7 (0–16)	5 (0–11)	0.012
Admission	Hemoglobin, g/dL	8.5 (4.2–11.5)	9.5 (3.6–15.7)	0.016
	LDH, U/L	1267 (558–3159)	2215 (258–5397)	0.0006
	Creatinine, mg/dL	0.66 (0.3–1.3)	3.3 (0.27–18.0)	< 0.0001
	Thrombocytes, × 10^9^/L	55 (14–275)	47 (8–356)	0.39
Minimum/maximum during course of disease	Hemoglobin, g/dL	6.0 (4.2–11.5)	6.2 (3.6–10.3)	0.38
	LDH, U/L	1831 (588–4260)	2706 (1297–7559)	< 0.0001
	Creatinine, mg/dL	0.8 (0.3–1.4)	6.0 (0.5–18.0)	< 0.0001
	Thrombocytes, × 10^9^/L	41 (14–237)	28 (5–166)	0.02
Discharge	Hemoglobin, g/dL	8.7 (6.6–12.1)	8.3 (6.4–12.9)^b^	0.44
	LDH, U/L	662 (330–1403)	474 (136–1834)^b^	0.001
	Creatinine, mg/dL	0.5 (0.2–1.0)	0.9 (0.2–7.4)^b,c^	< 0.0001
	Thrombocytes, × 10^9^/L	289 (65–675)	341 (61–837)^b^	0.66
	Duration of hospital stay, days	8 (3–12)	17 (4–188)^b^	< 0.0001

^a^Symptoms: diarrhea, vomiting, abdominal pain, fever

^b^In 88 survivors

^c^3 patients on dialysis at discharge excluded. *HUS*, hemolytic uremic syndrome; *LDH*, lactate dehydrogenase

**Fig. 1:**
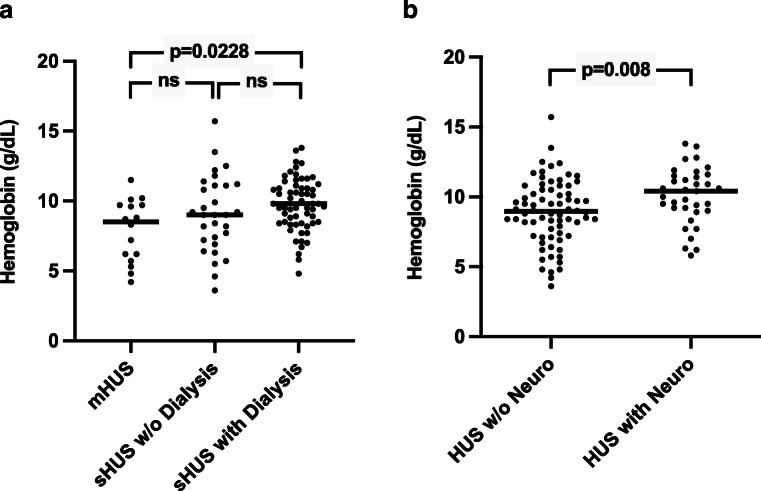
Hemoglobin level on admission in patients with mild (*n* = 16) vs. severe HUS (without (*n* = 30) and with dialysis (*n* = 61)) (**a**). Hemoglobin level on admission in patients without (*n* = 72) and with (*n* = 35) major neurological symptoms (neuro) (**b**). Individual values and median are shown

When the sHUS group was divided into patients with and without dialysis and those groups were compared to mHUS patients, there was a significant difference in the hemoglobin level at admission between mHUS and sHUS with dialysis groups (Fig. 1a). Thus, the highest hemoglobin levels on admission were associated with the most severe course of disease regarding kidney involvement.

At presentation and during the course of the disease, a higher LDH in the sHUS group compared to mHUS was observed (Table 1). However, the hemoglobin nadir and the hemoglobin at discharge did not significantly differ between patients with mHUS and sHUS (Table 1).

Ninety of one hundred seven patients (84%) received red blood cell transfusions. The proportion of patients receiving red blood cell transfusions was comparable between mHUS and sHUS patients (14/16 vs. 76/91 (OR 0.72 (95% CI 0.15–2.94), *p* = 0.69)). The total median number of red blood cell transfusions per patient was only slightly higher in the sHUS group (1 (0–2) vs. 1 (0–18), *p* = 0.042).

### Predictors of mild vs. severe HUS

**Fig. 2:**
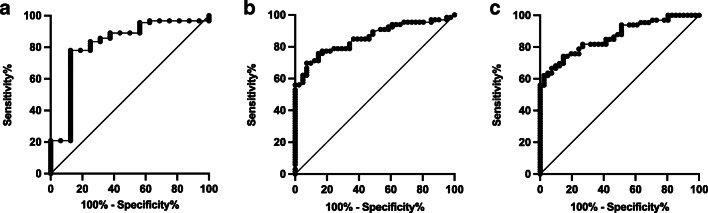
ROC curves the product of hemoglobin and lactate dehydrogenase (Hb (g/dL) × LDH (U/L)) on admission to predict development of severe vs. mild HUS (**a**) as well as ROC curves for creatinine (mg/dL) on admission (**b**) and hemoglobin (g/dL) + 2 × creatinine (mg/dL) on admission (**c**) to predict complicated HUS

Hemoglobin or LDH on admission alone was moderately predictive for sHUS (AUC 0.69 and AUC 0.76, respectively, data not shown). However, the product of hemoglobin and LDH (Hb (g/dL) × LDH (U/L)) on admission was able to distinguish subsequent sHUS from mHUS (AUC 0.82, 95% CI 0.69–0.94). A cutoff value of 13,302 had a sensitivity of 78.0% (95% CI 68.5–85.3%) and a specificity of 87.5% (95% CI 64.0–97.8%) (Fig. 2a).

### Predictors of complicated HUS

Patients with major neurological symptoms showed a significantly higher hemoglobin on admission than patients without neurological complications (Fig. 1b).

Patients with a complicated course of HUS (dialysis and/or major neurological symptoms) (*n* = 66) did not show a significantly higher hemoglobin on admission than patients without these complications (*n* = 41) (9.5 g/dL (4.8–13.8) vs. 8.8 g/dL (3.6–15.7), *p* = 0.06). However, serum creatinine (4.3 mg/dL (0.3–18.0) vs. 1.3 mg/dL (0.3–3.9), *p* < 0.0001) and LDH (2513 U/L (301–5397) vs. 1574 U/L (258–3159), *p* < 0.0001) on admission were significantly higher in patients with subsequently complicated HUS.

Therefore, serum creatinine (mg/dL) on admission was predictive for complicated HUS (AUC 0.86, 95% CI 0.79–0.93, Fig. 2b). Hb (g/dL) × LDH (U/L) on admission was moderately predictive (AUC 0.80, 95% CI 0.72–0.89, cutoff values not shown).

The previous established score hemoglobin (g/dL) + 2 × creatinine (mg/dL) on admission [18] showed a good predictive value for development of a complicated HUS (AUC 0.87, 95% CI 0.80–0.93, Fig. 2c). A cutoff value of 14.2 had a sensitivity of 80.3% (95% CI 69.2–88.1%) and a specificity of 73.2% (95% CI 58.1–84.3%).

## Discussion

This single-center, long-term study shows that patients with sHUS had higher hemoglobin and hematocrit levels on admission than patients with mHUS, indicating that patients with sHUS had profound hemoconcentration.

Furthermore, the highest values on admission occurred in patients with subsequent need for dialysis or with major neurological symptoms. Thus, hemoconcentration on admission is associated with disease activity and prognosis in STEC-HUS. However, we did not find a difference between groups for the combined end-point dialysis and/or major neurological complications (complicated HUS).

These findings could be confounded by the time point of admission since patients with mHUS were admitted later than patients with sHUS and the drop of the hemoglobin level could be more advanced due to ongoing hemolysis. In this case, a higher LDH might be expected. In contrast, LDH, which reflects hemolysis, was higher in sHUS patients vs. mHUS patients on admission. This should contribute to lowering the hemoglobin value in sHUS patients. However, LDH could also be increased due to multi-organ involvement in sHUS patients. The association of LDH with the more severe disease has been reported previously [14–16]. In our cohort, the product of hemoglobin and LDH on admission was a predictor of sHUS.

Interestingly, a later admission in milder cases of HUS was recently also observed by McKee et al. [15]. Alconcher et al. also reported an earlier admission in patients who died of STEC-HUS compared to survivors [13].

The score applied by Ardissino et al. (hemoglobin (g/dL) + 2 × creatinine (mg/dL)) was moderately predictive for a complicated course of HUS in their cohort. However, clinical definitions vary substantially between the study by Ardissino et al. and our definition of sHUS since in this series the complicated course of HUS was defined by death and the long-term kidney outcome (CKD stage 2–5) [18].

To validate the score established by Ardissino et al., we analyzed our cohort for its predictive value for complicated HUS defined by dialysis and/or major neurological symptoms. We found a good prediction with an AUC of 0.87. This was even better than in the recent study published by Lin et al. in 155 patients, in which they calculated a AUC of 0.71 using similar end-points to our study [21].

Incomplete or mild forms of STEC-HUS have been reported in up to 65% of the patients and no AKI was present upon admission in 14% of the patients [16, 26]. This has led to the discussion of the classical HUS criteria. However, data on the prevalence of mild forms of STEC-HUS in larger cohorts based on a clear case definition are lacking. To evaluate the prevalence of mHUS in our cohort, we defined kidney involvement in mHUS cases by a creatinine cutoff of < 1.5 mg/dL. This is equal to twice the maximum upper limit of serum creatinine across all age groups in our cohort and, since pre-HUS creatinine values are lacking in our patients, reflects the definition of AKI stage 1 according to the KDOQI guidelines most accurately [29]. In our cohort, a substantial number of patients (15%) had a mild form of HUS with only minimal kidney involvement and no major neurological symptoms during the course of the disease.

Interestingly, the minimum hemoglobin and the rate of transfusions did not differ between mild and severe HUS. However, the minimum hemoglobin could be confounded by the indication for transfusions. The number of transfusions per patient was slightly higher in the group with sHUS. In contrast, Cobenas et al. found a more pronounced association for the need for red blood cell transfusions with more severe kidney disease [14].

It remains unclear why some patients with STEC infection do not develop HUS at all and some are less prone to a more severe course of HUS. Unfortunately, the cause of the lower hemoconcentration in patients with mHUS could not be identified in our retrospective study. Probably, these patients were less affected in the gastrointestinal prodromal phase and had less fluid loss and/or a better oral fluid intake before admission. Even though our study was retrospective and interventional data is lacking, our data indirectly support the concept of infusion therapy/volume expansion in patients with STEC infection or HUS to prevent or treat intravascular volume depletion and improve organ perfusion and ultimately improve the course and outcome of STEC-HUS [5].

In conclusion, patients with subsequent sHUS have a higher degree of hemoconcentration on admission and sHUS can be predicted by Hb × LDH on admission. We could validate the predictive value of the score hemoglobin (g/dL) + 2 × creatinine (mg/dL) for complicated HUS. Since the correction of hemoconcentration seems to be a simple and effective therapeutic measure, further prospective studies combining treatment and rheological data are needed to strengthen this concept.

## Supplementary Information

ESM 1 (PPTX 114 kb).

## Abbreviations

AKI: acute kidney injury
CKD: chronic kidney disease
HUS: hemolytic uremic syndrome
Hb: hemoglobin
LDH: lactate dehydrogenase
mHUS: mild hemolytic uremic syndrome
KRT: kidney replacement therapy
sHUS: severe hemolytic uremic syndrome
STEC: Shiga toxin-producing *Escherichia coli*
Stx: Shiga toxin
