# Supplementary material for: Hemoconcentration and predictors in Shiga toxin-producing E. coli-hemolytic uremic syndrome (STEC-HUS)
Source: Pediatr Nephrol. 2021 May 27;36(11):3777–83. doi: 10.1007/s00467-021-05108-6 (PMC8497454; doi:10.1007/s00467-021-05108-6)
Supplement: Supplementary file 1 — (PPTX 114 kb). [file 467_2021_5108_MOESM1_ESM.pptx]

## Slide 1
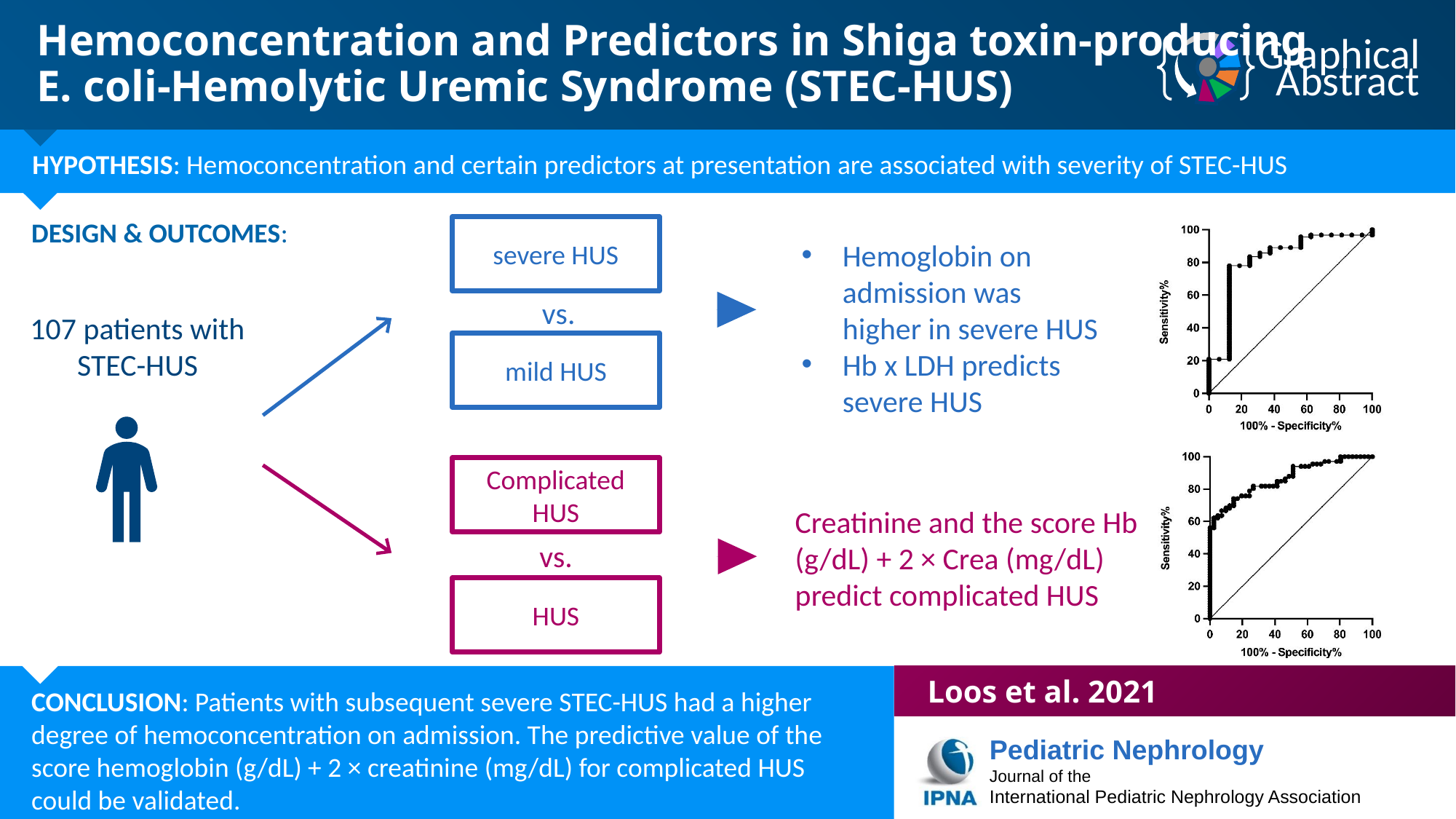

Hemoconcentration and Predictors in Shiga toxin-producing
E. coli-Hemolytic Uremic Syndrome (STEC-HUS)
HYPOTHESIS: Hemoconcentration and certain predictors at presentation are associated with severity of STEC-HUS
DESIGN & OUTCOMES:
severe HUS
Hemoglobin on admission was higher in severe HUS
Hb x LDH predicts severe HUS
vs.
107 patients with
STEC-HUS
mild HUS
Complicated HUS
Creatinine and the score Hb (g/dL) + 2 × Crea (mg/dL) predict complicated HUS
vs.
HUS
Loos et al. 2021
CONCLUSION: Patients with subsequent severe STEC-HUS had a higher degree of hemoconcentration on admission. The predictive value of the score hemoglobin (g/dL) + 2 × creatinine (mg/dL) for complicated HUS could be validated.
